# Bentonite-supplemented diets improved fish performance ammonia excretion haemato-biochemical analyses immunity antioxidants and histological characteristics of European seabass *Dicentrarchus labrax*

**DOI:** 10.1038/s41598-024-63936-6

**Published:** 2024-06-15

**Authors:** Alaa A. El-Dahhar, Ashraf. I. G. Elhetawy, Wael M. A. Refaey, Samy Y. El-Zaeem, Elsayed H. Elebiary, Ayman M. Lotfy, Mohamed M. Abdel-Rahim

**Affiliations:** 1 Animal and Fish Production Department, Faculty of Agriculture, Saba-Basha, Alexandria University, Alexandria, Egypt; 2 Aquaculture Division, National Institute of Oceanography and Fisheries, NIOF, Cairo, Egypt

**Keywords:** *Dicentrarchus labrax*, Bentonite feed additive, Growth performances, Ammonia excretion, Immune response, Health of the intestine and liver, Zoology, Ecology, Ocean sciences

## Abstract

The purpose of this research was to examine the potential effects of bentonite (BN) supplemented diets on growth, feed utilization, blood biochemistry, and histomorphology of *Dicentrarchus labrax*. Six treatments in triplicate were tested: B0, B0.5, B1.0, B1.5, B3.0, and B4.5, which represented fish groups fed diets supplemented with 0, 0.5, 1, 1.5, 3, and 4.5% BN, respectively. For 84 days, juveniles’ seabass (initial weight = 32.73 g) were fed diets containing 46% protein, three times daily at 3% of body weight. With a 5% daily water exchange, underground seawater (32 ppt) was used. Findings revealed significant improvements in water quality (TAN and NH3), growth (FW, WG and SGR) and feed utilization (FCR, PER and PPV) in fish fed BN-supplemented diets, with the best values in favor of the B1.5 group. Additional enhancements in kidney function indicators (urea and uric acid) and liver enzymes were observed in fish of the BN-treated groups along with a decrease in cholesterol level in the B1.5 group. Further improvements in fish innate immunity (hemoglobin, red blood cells, glucose, total protein, globulin, and immunoglobulin IgM), antioxidant activity (total antioxidative capacity and catalase), and decreased cortisol levels in fish of the BN-treated groups. Histological examinations of the anterior and posterior intestines and liver in groups B1.5 and B3 revealed the healthiest organs. This study recommends BN at a concentration of 1.5% as a feed additive in the *Dicentrarchus labrax* diet.

## Introduction

Nowadays, the aquaculture industry is critical due to its vital role in supplying approximately half of the world's animal protein needs^1,2^ and participating in the protection of imperiled species like sturgeons^3^. The prevalence of aquaculture worldwide, especially in the form of intensive and super intensive farms, and their deleterious discharges of organic and inorganic metabolites have prompted efforts to mitigate these damages and improve water quality in the surrounding environment^2,4^. Due to their unique structure, chemical composition, exchangeable ions, and particle size, a variety of clay minerals are now used as additives in the water where aquatic animals are raised to improve water quality and promote the removal of organic compounds^5–7^).

Clay minerals have been used in animal/fish feed due to their sorption/absorption properties, which contribute greatly to the health of organisms because they bind to harmful compounds and remove them from the body^8^, play a crucial role in detoxifying anti-nutritional compounds in food and relieving gastrointestinal ailments, as well as acting as mycotoxin adsorbents^9^. Bentonites are clays that naturally occur from silicate minerals and have been used to eliminate ammonium from fish tanks due to their lower cost, greater market availability, and superior selectivity^10^. They belong to the stratum troupe of aluminosilicates with extending frames that characterize their great absorbency and swelling^7^. As a mineral generated from dust, BN has an additional significant non-nutritive component that is employed as a powerful binding agent and adsorbent for mycotoxins, enzymes, and pathogenic microorganisms in animal feed and the intestines of animals^11–13^. BN clays could enhance the sorption exchange capacity of the acid-salt-soda activation string ^7,14^. Two types of bentonites are known: sodium BN, which typically has a highly turbulent type and is derived from volcanic ash that precipitated in marine environments, and calcium BN, which has a low-tumescence type and is derived from volcanic ash that precipitated in freshwater environments^6,10^.

Previous studies reported that BN has a good influence on fish performance when employed as a feed additive. According to Ellis et al.^15^ the addition of sodium BN to the diet of trout significantly reduces aflatoxin levels in the digestive tract. In addition, Ayoola^12^ observed that the addition of BN to various rations greatly increased the growth, feed consumption, specific growth rate, red blood cells, and hematocrits of the African catfish, *Clarias gariepinus*. Furthermore, the growth, survival, dietary consumption, and hematological indicators of the hybrid grouper (*Epinephelus fuscoguttatus* x *Epinephelus lanceolatus*), fed diets supplemented with varying concentrations of sodium BN for 8 weeks, were significantly enhanced in comparison to the control group^16^. In addition, the previous data demonstrated that Nile tilapia fed lead-contaminated diets exhibited a significant decrease in growth, blood parameters compared to fish fed the same diets with BN as a feed additive^17^. Also, tilapia fed aflatoxin-contaminated feed in the presence of BN had much higher concentrations of albumin, total protein, erythrocytes, and hemoglobin, and lead residues were significantly lower^17^.

The European seabass D*. labrax* is an important aquaculture species in Europe and the Mediterranean region, with a 2.9% share of the global aquaculture harvest in 2020^1^. The global harvest of *D. labrax* has continuously increased from approximately 60,000 tonnes (T) in 2003 to 243,900 T in 2020^1^. Egypt ranks third among the world's largest producers with a total of 34,477 T, after Turkey and Greece^1,18^. However, no studies have been published on the use of BN clay as a feed additive for the most important marine species cultivated in Egypt, such as gilthead seabream *Sparus aurata* and *D. labrax*^1,19^. Domestic BN has a high capacity for cation exchange, binding capacity, plasticity, impermeability, hydration, swelling, thixotropy, and a tendency to react with organic chemicals^20–22^, can protect the intestinal tract by rapidly binding toxins in the gut, in addition to providing the appropriate minerals to improve the nutritional efficiency, growth, and overall health of *D. labrax*. This study evaluates the potential ameliorative influences of using BN clay as a feed additive on the growth, immunology, antioxidants, and histology of *D. labrax* grown in groundwater.

## Materials and methods

At El-Max Station for Applied Research of NIOF, Alexandria, Egypt, this experiment was carried out in cooperation with Alexandria University, Faculty of Agriculture (Saba Basha)—Department of Animal and Fish Production.

### Experimental design

Six treatments were tested in triplicate to determine the potential benefits of adding raw BN to *D. labrax* aquafeed, as follows: (B0) = control without BN addition, (B0.5) = adding BN at a level of 0.5%, (B1) = adding BN at a level of BN 1% , (B1.5) = adding BN at a level of BN 1.5%, (B3) = adding BN at a level of BN 3%, and (B4.5) = adding BN at a level of BN 4.5%. The levels studied were chosen based on earlier research^17,23^. The duration of the experimental period was 84 days. During the experiment period, *D. labrax* fingerlings were raised in underground saltwater with a salinity of 32 ppt.

### Experimental fish and rearing techniques

The *D. labrax* juveniles used in this study were obtained from the Kilo-21 marine hatchery (GAFRD), Alexandria, Egypt. The juveniles were produced using Mediterranean Sea water. After arriving the experimental station, fish were acclimatized to the new water conditions for two weeks and fed commercial pelleted feed (46/16) protein/fat, prior to the start of the experiment. One hundred and eighty juvenile *D. labrax* L. with an average initial weight of 32.73 ± 0.04g/fish, and an averae initial length of 13.93 ± 0.2 cm/fish, were used. This experiment utilized six cement tanks (each 3m × 8m and containing 24 m^3^ water) and eighteen net enclosures (experimental hapa: 1m x 1m x 1m, each containing 0.5 m^3^ of water). Fish were placed at a density of 10 fish/hapa, and every three hapas were housed in a cement tank, representing one treatment.

### Diet preparation and feeding regime

Six levels of local BN (0, 0.5, 1, 1.5, 3, and 4.5% BN /kg feed) were supplemented with a commercial diet used for marine carnivorous fish. The abbreviations B0, B0.5, B1, B1.5, B3, and B4.5 represent diets supplemented with 0, 0.5, 1, 1.5, 3, and 4.5% BN per kilogram of diet, respectively. The desired amount of very fine BN was carefully mixed with the basic diet, which had been finely ground (Moulinex stand mixer—Qa205127). Following the addition of BN and hot water, the diets were pressed using an electric kitchen meat grinder (Moulinex 1600 W, France). The feed strands were then dried at 45 degrees Celsius for 12 h. Using various feed sieves, the pellets were sieved to achieve the desired particle sizes. The pellets were packed and kept at a temperature of − 20°C until use. The fish were fed to satiation four times a day, six days a week. Table 1 shows the chemical composition of the commercial diet and BN from Egypt's Central Eastern Desert. High purity BN purchased from an Egyptian distributor and collected from El Qoseir area in the central eastern desert, Egypt, located on the coast of the Red Sea, was used in the present study. Dardir et al.^22^ investigated the physicochemical properties of the BN used in the experiment and determined that the average particle size is 8 um, the average pore diameter is 12.6 nm, the specific surface area is 102.5 m2/g, and the cation exchange capacity is 127 meq/100 g.

**Table 1:** Chemical composition of commercial feed and raw bentonite in central eastern desert, Egypt.

Chemical analysis of commercial feed											
Composition	Dry Matter		Crude Protein	Ether extract	Fibers	NFE	Ash	Gross energy (kcal/100gm)		N:C ratio (mg CP: Kcal)	
%	92		46	16	3.03	19.99	14.98	492.64		93.37	

Chemical composition of raw bentonite*											
Chemical compound	SiO2	Al2O3	MgO	Fe2O3	Na2O	K2O	CaO	TiO2	P2O5	MnO	LOI
% in raw bentonite	54.53	17.25	3.16	4.67	1.87	0.75	1.14	0.03	0.04	0.02	16.52

Source: *Dardir et al.^22^.

### Water quality analyzes

Throughout the experiment, temperature, dissolved oxygen, pH, total ammonia nitrogen (TAN), and un-ionized ammonia (NH3) were monitored twice weekly for all treatments. SensoDirect 150 (Multiparameter, portable photometer) was used to measure ph/Redox, conductivity, dissolved oxygen, TDS, and temperature (°C) for water analysis. TAN was determined using the Hanna HI-97715 model (portable photometer, Medium-range ammonia, Hanna Instruments, Romania). Using recorded data for pH, temperature, salinity, and TAN, NH3 was calculated.

### Fish and feed analytical methods

The chemical composition of both commercial diet and fish was performed at the start of the experiment using ten fish, and at the end with nine fish per treatment (3 per replicate) to estimate the moisture, crude protein, crude fat, fiber and seabass ash, according to Cunniff, Pand Washington^24^.

### Growth performance and feed utilization indices

The fish were collected, counted, and weighed at the end of the experiment. The following growth performance and feed utilization measurements were determined:

Weight gain (g/fish), WG = Wt − W0; Average daily gain, ADG (g∕fish∕day) = Wt − W0∕days; Specific growth rate (%∕day): SGR = 100 × (lnWt − lnW0)∕days, where: W0: initial fish weight (g); Wt: final fish weight (g); ln: natural logarithm; Survival rate (%) = 100 x (Final number of fish ∕ initial number of fish); Condition factor = 100 × ((BW (g)/L^3^ (cm)); Condition factor (CF, g/cm^3^) = final body weight (g) / final body length (cm)^3^.

Feed conversion ratio (FCR)-based on dry matter (DM) = feed intake (g) as DM ∕ weight gain (g); Protein efficiency ratio PER = weight gain (g) ∕protein intake (g); Protein productive value PPV% = 100 × (protein gain (g) ∕protein intake (g)); Energy gain (EG)Kcal = Et − E0, where: Et: Energy content in the fish carcass (Kcal) at the end, E0: Energy content in the fish carcass (Kcal) at the start; Energy utilization (EU%) = 100 x (Energy gain (kcal) ∕Energy intake (kcal)). Viscerosomatic index (VSI, %) = 100 × (viscera weight (g) / fish body weight (g)); Hepatosomatic index (HSI, %) = 100 × (liver weight (g) / fish body weight (g));

### Hematological analysis

#### Blood sampling

At the end of the experiment, blood samples were drawn from the caudal vertebral vein of an anesthetized fish with MS222 at 100 mg/L of three animals per triplicate and nine fish per treatment.

#### Blood analysis

An automatic blood cell counter (Exigo-Vet., Boule Medical AB Inc., Stockholm, Sweden) was used to measure the number of red blood cells (RBCs), hemoglobin concentration, packed cell volume (PCV), and total and differential white blood cells (WBCs)^25^ .

#### Serum biochemical analysis

Serum triglyceride (TG) level was analyzed using the TG quantification Kit (MAK266, Sigma-Aldrich, St Louis, MO, USA). In this assay, TG is converted to free fatty acids and glycerol^26^. The total cholesterol (CHO) concentration is determined by free CHO and cholesteryl esters enzyme assays^26^. Total protein (TP) and albumin (ALB) were determined using commercially available kits (Bio-diagnostics, Giza, Egypt), and the difference between TP and ALB content was reported as globulin (GLO) content^27^. Methods described by Trinder^28^ were used to evaluate serum glucose (GLU). Serum total immunoglobulin (IgM) was determined by precipitating Ig with polyethylene glycol and subtracting the initial and final total protein according to Siwicki^29^. Serum digestive enzymes (amylase and lipase) were evaluated according to Zamani et al. (2009). Aspartate aminotransferase (AST) and alanine aminotransferase (ALT) were assayed as described by Bergmeyer, et al.^30^ with 0.2 M DL-aspartic acid and 20 mM L-ketoglutarate as substrate and 0.2 M DL-alanine and 2 mM L ketoglutarate, respectively. Alkaline phosphatase (ALP) was assayed as described by Bessey et al.^31^. Using a colorimetric method of Heinegrd and Tiderstrom^32^, the serum creatinine (CRE) concentration was determined. The concentration of uric acid (UA) was determined using the method described by Whitehead et al.^33^. Cortisol (CORT) activity was assayed according to Foster and Dunn^34^. The catalase (CAT) enzyme was measured using Aebi's^35^ method. Using an automatic biochemical analyzer, the activities of total antioxidant capacity (TAC) were assessed (Hitachi 7600D, Hitachi, Tokyo, Japan) according to the method of Koracevic et al.^36^. Glutathione peroxidase (GPx) levels were detected using the respective techniques of Paglia and Valentine^37^. Malonaldehyde (MDA) levels were determined using Uchiyama and Mihara's^38^ methodology.

#### Histology of liver, and intestine

Liver and intestine (anterior and posterior) samples were obtained using three fish per replicate and nine fish per treatment, from all groups, then fixed in 10% neutral buffered formalin. An automated tissue processor was used to process the formalin-preserved intestinal and hepatic tissues from seabass fish. An automated tissue processor was used to process the formalin-preserved intestinal and hepatic tissues from seabass. The procedure began with a two-step fixation and dehydration. The tissue was fixed by immersion in 10% buffered formalin for 48 h, then removing the fixative in distilled water for 30 min. The tissues were then dehydrated by passing through a graded sequence of alcohol (70%, 90%, and 100%). The tissue was first subjected to 70% alcohol for 120 min, then 90% alcohol for 90 min, and then to two cycles of one hour of absolute alcohol. Following dehydration, the samples were cleared in numerous changes of xylene. It involved immersing the tissue for one hour in a mixture of 50% alcohol and 50% xylene, followed by one and a half hours in pure xylene. After that, the samples were impregnated with molten paraffin wax, imbedded, and blacked out. On a Leica Rotary Microtome (RM 2145, Leica Microsystems, Wetzlar, Germany), paraffin longitudinal Sects. (4–5 um) were cut and mounted on glass slides. According to Feldman and Wolfe (2014), slides are then commonly stained with Hematoxylin and Eosin (H&E). Image J analysis software (National Institutes of Health, MD, USA) was used for histomorphometric analysis, and the length, width of intestinal villi, and Crypt's length, as well as muscle layer thickness, were measured.

### Statistical analyses

The results are expressed as mean SEM. The data meet the homogeneity of variance assumption. Data were statistically analyzed with a one-way analysis of variance (ANOVA) using SPSS software (Standard Version 26.0 SPSS Inc. Chicago, Illinois). Duncan's multiple range test was utilized to compare differences among treatment means at the *P* < 0.05 level.

### Ethical declaration

The research experiment was approved by the University of Alexandria, Institutional Animal Care and Use Committee, IACUC approval AU19/22/10/20/3/26, Alexandria University, Egypt. The authors confirm that all methods were performed in accordance with the relevant guidelines and regulations, and that the study is reported in accordance with the ARRIVE guidelines.

## Results

### Influences of dietary BN on water quality

**Figure 1:**
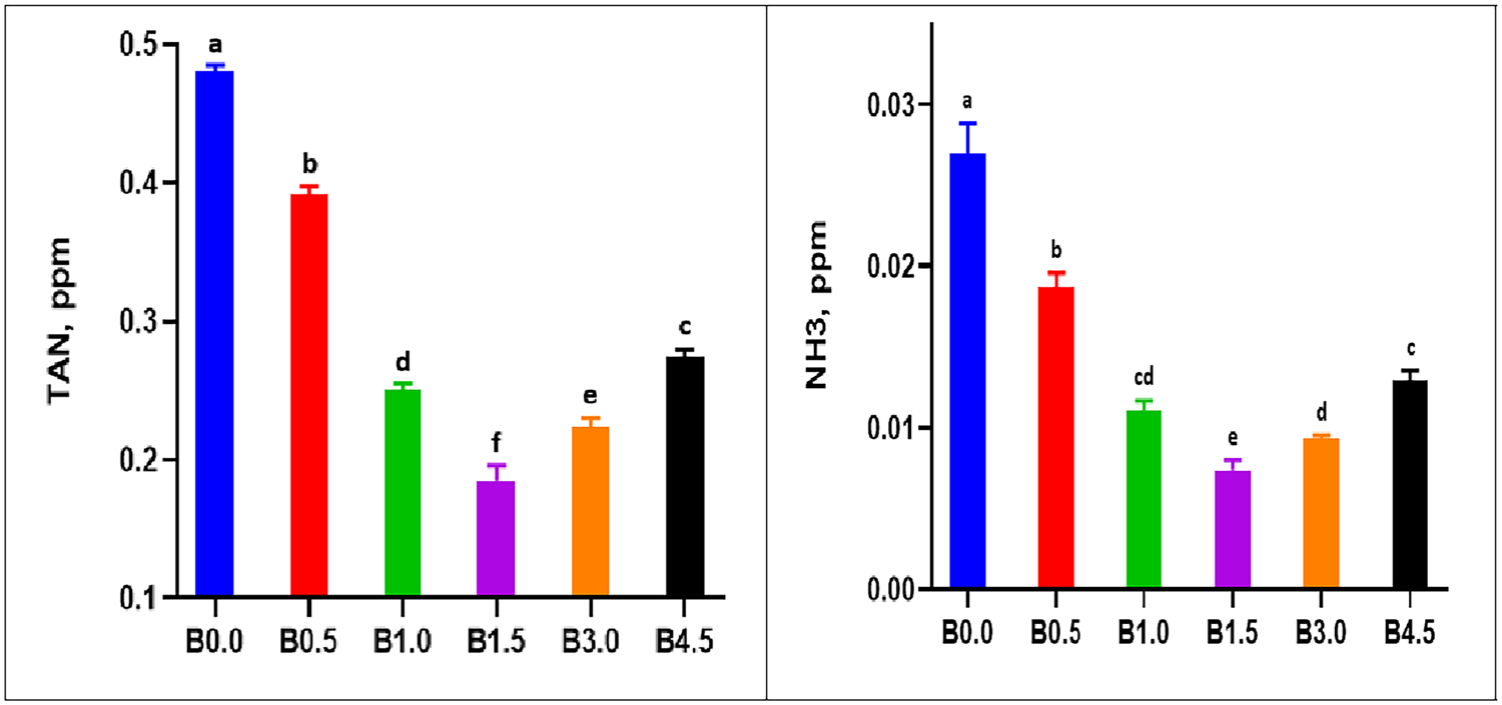
Total ammonia nitrogen (TAN) and unionized ammonia (NH3) in seabass ponds fed with different levels of bentonite. Where B0 = 0% bentonite, B0.5 = 0.5% bentonite, B1 = 1% bentonite, B1.5 = 1.5% bentonite, B3.0 = 3.0% bentonite, and B4.5 = 4.5% bentonite.

Throughout the period of the experiment, all water quality parameters remained within acceptable ranges (Boyd and Tucker 2012). The average water temperature, dissolved oxygen concentration, nitrite concentration and pH were 25.5°C, 6.1 mg/L, 0.027 mg/L and 7.92, respectively. During the 12-week test period, total TAN and NH3 concentrations changed significantly (*P* < 0.05) among the six groups, as shown in Fig. 1. The highest concentrations of TAN and NH3 were found in the B0 group, while the lowest levels were found in group B1.5. Additionally, the TNA and NH3 concentrations in all groups treated with BN were significantly lower than in the B0 group.

## Growth performance and feed utilization indices of *D. labrax*

The level of BN in the diet had a significant effect on growth parameters, as determined by a mean ANOVA (Table 2). The growth indices of the fish fed the BN-containing diets were significantly (*P* < 0.05) superior to those fed a control diet. At the end of the experiment, the fish in group B 1.5 grew significantly more and had enhanced growth characteristics (higher FW, WG, SGR and CF) than those fed the control diet or other diets supplemented with BN. Overall, the addition of BN improved the performance of the fish in all treated groups, with group B1.5 showing the highest significant increase in SGR and FW, followed by groups B1 and B3 with no significant differences between them. The feed utilization parameters of *D. labrax* fingerlings are presented in (Table 2). FCR varied significantly among the six treatments, with the best value (1.43) recorded for fish in group B1.5 and the worst value (2.03) recorded for fish in group B0. Increasing the amount of BN in the diet to 3% and 4.5% adversely affected the FCR value. The values of the indices PER, PPV, EG, and EU% formed a fluctuating curve, with group B0 being the starting point (lowest value) and B 4.5 being the endpoint (lowest value), while group B1.5 represented the top of the curve.

**Table 2:** Growth performance, condition factor and survival of *Dicentrarchus labrax* fingerlings as affected by dietary bentonite.

Parameters	Treatments					
	B0	B0.5	B1.0	B1.5	B3.0	B4.5
Growth parameters						
FW (g fish-^1^)^1^	90.57 ± 0.73^d^	95.67 ± 0.66^c^	105.13 ± 0.58^b^	114.96 ± 0.68^a^	105.13 ± 0.70^b^	94.43 ± 0.52^c^
WG (g fish-^1^)	57.77 ± 0.82^d^	62.87 ± 0.77^c^	72.47 ± 0.52^b^	82.16 ± 0.62^a^	72.47 ± 0.52^b^	61.77 ± 0.50^c^
ADG (g fish/day)	0.688 ± 0.01^d^	0.748 ± 0.01^c^	0.863 ± 0.01^b^	0.978 ± 0.01^a^	0.863 ± 0.01^b^	0.735 ± 0.01^c^
SGR (% day)	1.209 ± 0.13^d^	1.274 ± 0.01^c^	1.392 ± 0.01^b^	1.493 ± 0.01^a^	1.391 ± 0.01^b^	1.264 ± 0.01^c^
CF	0.980 ± 0.05^b^	1.083 ± 0.06^ab^	1.077 ± 0.04^ab^	1.131 ± 0.02^a^	1.056 ± 0.03^ab^	1.032 ± 0.01^ab^
Survival (%)	100	100	100	100	100	100
Feed utilization						
FCR	2.03 ± 0.04^a^	1.86 ± 0.03^b^	1.62 ± 0.01^c^	1.43 ± 0.01^d^	1.62 ± 0.01^c^	1.90 ± 0.02^b^
PER	1.07 ± .02^d^	1.17 ± 0.02^c^	1.34 ± 0.01^b^	1.52 ± 0.01^a^	1.34 ± 0.01^b^	1.14 ± 0.01^c^
PPV, %	18.32 ± 0.60^d^	19.71 ± 0.48^c^	23.34 ± 0.23^b^	26.38 ± 0.47^a^	23.53 ± 0.10^b^	19.66 ± 0.23^c^
Energy gain (EG), Kcal	110.14 ± 2.71^d^	117.22 ± 2.43^c^	139.74 ± 1.49^b^	155.59 ± 2.44^a^	138.89 ± 0.95^b^	117.63 ± 1.86^c^
Energy utilization, EU%	19.09 ± 0.55^d^	20.36 ± 0.45^cd^	24.16 ± 0.23^b^	26.96 ± 0.27^a^	24.03 ± 0.08^b^	21.77 ± 1.56^c^
Biometric parameters^2^						
HSI (%)	1.79 ± 0.21^bc^	1.89 ± 0.16^b^	2.27 ± 0.16^a^	2.08 ± 0.01^ab^	1.83 ± 0.13^b^	1.81 ± 0.07^b^
VSI (%)	8.58 ± 0.82^d^	8.99 ± 0.80^c^	11.35 ± 0.70^a^	10.81 ± 0.09^b^	8.43 ± 0.27^d^	7.72 ± 0.65^e^
DL/TL (%)	66.48 ± 12.87	75.80 ± 8.91^d^	89.60 ± 4.62^b^	92.67 ± 1.26^a^	84.76 ± 1.11^c^	90.83 ± 1.32^a^

^1^Initial body weight = 32.73 ± 0.04 gm; ^2^DL = Digestive length, TL = Total length; values are expressed as the mean ± SE; Different letters in the same row indicate significant differences among the treatments (*P* ≤ 0.05). Where CF is Condition factor, B0 = 0% bentonite, B0.5 = 0.5% bentonite, B1 = 1% bentonite, B1.5 = 1.5% bentonite, B3.0 = 3.0% bentonite, and B4.5 = 4.5% bentonite.

### Whole-body chemical composition

The effects of dietary BN levels on the chemical composition of *D. labrax*'s whole body are described in Table 3. Statistical analysis found that the proportions of protein and dry matter in flesh did not vary significantly between the six treatments. Furthermore, there were no significant variations (*P* < 0.05) variations in the fat content of fish carcasses, between the BN-treated groups and the control, except for fish from the B3 group. Regarding fish body ash, the addition of BN up to 1% reduced the ash content in the carcass of the fish that recorded the lowest value with fish of the B1 group, while an increase in the ash level occurred with increasing the dietary BN to 1.5, 3, and 4.5%, as these groups did not differ significantly from the control.

**Table 3:** Carcass composition of *Dicentrarchus labrax* fingerlings as affected by dietary bentonite.

Carcass composition	Initial	Final					
		B0.0	B0.5	B1.0	B1.5	B3.0	B4.5
Dry matter, %	33.78 ± 0.17	33.62 ± 0.25	33.20 ± 0.22	33.45 ± 0.22	33.32 ± 0.25	33.88 ± 0.15	33.38 ± 0.17
Protein, %	56.53 ± 0.35	53.9 ± 0.11	53.18 ± 0.27	53.69 ± 0.22	53.57 ± 0.21	53.28 ± 0.18	53.48 ± 0.34
Ether extract, %	26.01 ± 0.18	28.35 ± 0.09^ab^	28.17 ± 0.15^ab^	28.77 ± 0.20^a^	28.32 ± 0.16^ab^	28.00 ± 0.15^b^	28.51 ± 0.33^ab^
Ash, %	16.33 ± 0.22	18.19 ± 0.39^a^	17.41 ± 0.19^b^	16.72 ± 030^c^	17.76 ± 0.07^ab^	18.29 ± 0.28^a^	17.64 ± 0.05^ab^
Carcass-energy, Kcal/100gm	–	567.1 ± 0.75^bc^	565.9 ± 0.71^bc^	574.4 ± 2.63^a^	569.4 ± 0.44^abc^	564.8 ± 2.47^c^	570.8 ± 1.38^ab^

Values are expressed as the mean ± SE. Different letters in the same row indicate significant differences among the treatments (*P* ≤ 0.05). Where B0 = 0% bentonite, B0.5 = 0.5% bentonite, B1 = 1% bentonite, B1.5 = 1.5% bentonite, B3.0 = 3.0% bentonite, and B4.5 = 4.5% bentonite.

### Effects on blood hematological and biochemical parameters

The addition of BN to fish diets significantly (*P* < 0.05) improved the blood biochemical parameters of *D. labrax* (Table 4). Compared to the control diet, fish-fed BN-supplemented diets demonstrated higher values of blood parameters such as hemoglobin, RBCs and WBCs. Supplementation of the *D. labrax* diet with BN at various levels resulted in significant changes in the serum lipid profile. Significant decreases in CHO, HDL, and LDL levels were recorded for B0.5 and B1.5 compared to B0. Groups B0 and B0.5 had the lowest TG value. Groups B3 and B4.5 had significantly (*P* < 0.05) higher values of CHO, TG and HDL, while the B1 group had the highest level of LDL (Table 5).

**Table 4:** Effect of different levels of dietary bentonite on blood biochemical analyses of European seabass (*Dicentrarchus labrax*).

Blood Parameters	Treatments					
	B0.0	B0.5	B1.0	B1.5	B3.0	B4.5
Hb (g/100ml)	12.05^d^	13.55^c^	14.15^b^	14.80^a^	14.10^b^	13.35^c^
RBCs (× 10/mm^3^)	3.05^d^	4.31^ab^	4.32^ab^	4.37^a^	4.24^b^	3.86^c^
WBCs (× 10^3^/mm^3^)	124.0^b^	121.5^c^	117.2^d^	115.3^e^	119.65^c^	126.50^a^
Lymphocyte (%)	71.5^e^	76.0^d^	74.0^d^	81.95^b^	79.15^c^	88.15^a^
Neutrophils (%)	15.5^b^	15.5^b^	20.5^a^	11.1^d^	14.1^c^	7.05^e^
Monocyte (%)	9.0^a^	5.0^b^	3.0^c^	5.0^b^	4.0^bc^	4.0^bc^
Eosinophil (%)	5.5^a^	3.0^b^	2.0^c^	2.0^c^	3.0^b^	1.0^d^
Hematocrit (HCT) (%)	53.05^ab^	50.50^abc^	48.90^bc^	44.20^cd^	56.70^a^	38.85^c^
MCV (µm^3^/cell)	121.50^c^	123.50^c^	132.15^ab^	129.80^b^	134.20^a^	130.55^b^
MCH (pg/cell)	33.50^d^	36.10^c^	39.55^a^	36.75^b^	31.30^e^	33.60^d^
MCHC (%)	27.60^e^	28.90^d^	31.55^c^	35.55^a^	23.40^f^	33.70^b^
RDW-CV	14.60^a^	13.55^c^	13.65^c^	14.15^b^	14.90^a^	9.30^d^
RDW-SD	61.85^b^	51.50^c^	45.25^e^	50.85^c^	67.75^a^	48.85^d^
PLT- (#/mcL)	26.00^a^	11.50^cd^	12.90^c^	14.95^b^	8.15^e^	10.30^d^

The values with a different superscript in the same row are significantly different (*P* ≤ 0.05). Where B0 = 0% bentonite, B0.5 = 0.5% bentonite, B1 = 1% bentonite, B1.5 = 1.5% bentonite, B3.0 = 3.0% bentonite, and B4.5 = 4.5% bentonite.

**Table 5:** Influences of dietary bentonite on serum lipid profile of European seabass (*Dicentrarchus labrax*).

Serum lipid parameters	Treatments					
	B0.0	B0.5	B1.0	B1.5	B3.0	B4.5
Cholesterol (CHO)	281.0 ± 7.0^b^	199.0 ± 3.0^c^	299.0 ± 12.0^ab^	190.0 ± 3.0^c^	319.0 ± 8.0^a^	325.0 ± 8.0^a^
Triglyceride (TG)	208.0 ± 3.0^c^	218.5 ± 9.5^c^	296.0 ± 3.0^b^	279.5 ± 1.5^b^	325.0 ± 9.0^a^	323.5 ± 5.5^a^
HDL	94.5 ± 0.5^c^	62.5 ± 1.5^d^	95.5 ± 0.5^c^	58.5 ± 1.5^d^	126.5 ± 5.5^b^	138.0 ± 5.0^a^
LDL	119.5 ± 1.5^bc^	85.0 ± 2.0^d^	132.5 ± 1.5^a^	75.0 ± 1.0^d^	126.5 ± 2.5^ab^	114.5 ± 6.5^c^

The values with a different superscript in the same row are significantly different (*P* ≤ 0.05). Where B0 = 0% bentonite, B0.5 = 0.5% bentonite, B1 = 1% bentonite, B1.5 = 1.5% bentonite, B3.0 = 3.0% bentonite, and B4.5 = 4.5% bentonite.

The addition of BN to the diet had a significant effect (*P* < 0.05) effect on the indicators of kidney function of *D. labrax* (Table 6). The levels of urea and UA decreased significantly in the B1 and B1.5 groups compared to the B0 group. Meanwhile, the addition of BN to the seabass diet did not affect CRE levels while the B4.5 group had a significantly higher ammonia value than the B0 group. Serum liver enzymes (ALT, AST, and ALP) showed significant decreases (*P* < 0.05) in BN-treated fish diets compared to those fed the control diet. The lowest AST values were observed in the B0.5, B1, B1.5, and B3 groups, respectively, while the lowest ALT value was recorded in the B0.5, B1 and B1.5 groups, with no significant differences between them. Except for the B4.5 group, ALP levels decreased significantly in all groups treated with BN compared to the control, with the lowest value in the B0.5 group.

**Table 6:** Influences of dietary bentonite on kidney enzymes of European seabass (*Dicentrarchus labrax*).

Serum kidney parameters	Treatments					
	B0.0	B0.5	B1.0	B1.5	B3.0	B4.5
Urea	25.5 ± 0.86^a^	23.5 ± 0.87^ab^	18.5 ± 0.87^c^	15.0 ± 0.58^d^	15.0 ± 0.85^d^	21.5 ± 0.86^b^
Creatinine	0.73 ± 0.01	0.64 ± 0.08	0.59 ± 0.01	0.65 ± 0.01	0.62 ± 0.01	0.68 ± 0.01
Uric acid	1.67 ± 0.07^a^	1.67 ± 0.03^a^	1.16 ± 0.03^b^	1.10 ± 0.04^b^	1.61 ± 0.03^a^	1.54 ± 0.06^a^
Ammonia	58.0 ± 0.58^b^	60.0 ± 1.15^ab^	59.0 ± 3.46^ab^	59.5 ± 0.87^ab^	57.0 ± 1.15^b^	65.0 ± 2.31^a^

The values with a different superscript in the same row are significantly different (*P* ≤ 0.05). Where B0 = 0% bentonite, B0.5 = 0.5% bentonite, B1 = 1% bentonite, B1.5 = 1.5% bentonite, B3.0 = 3.0% bentonite, and B4.5 = 4.5% bentonite.

**Figure 2:**
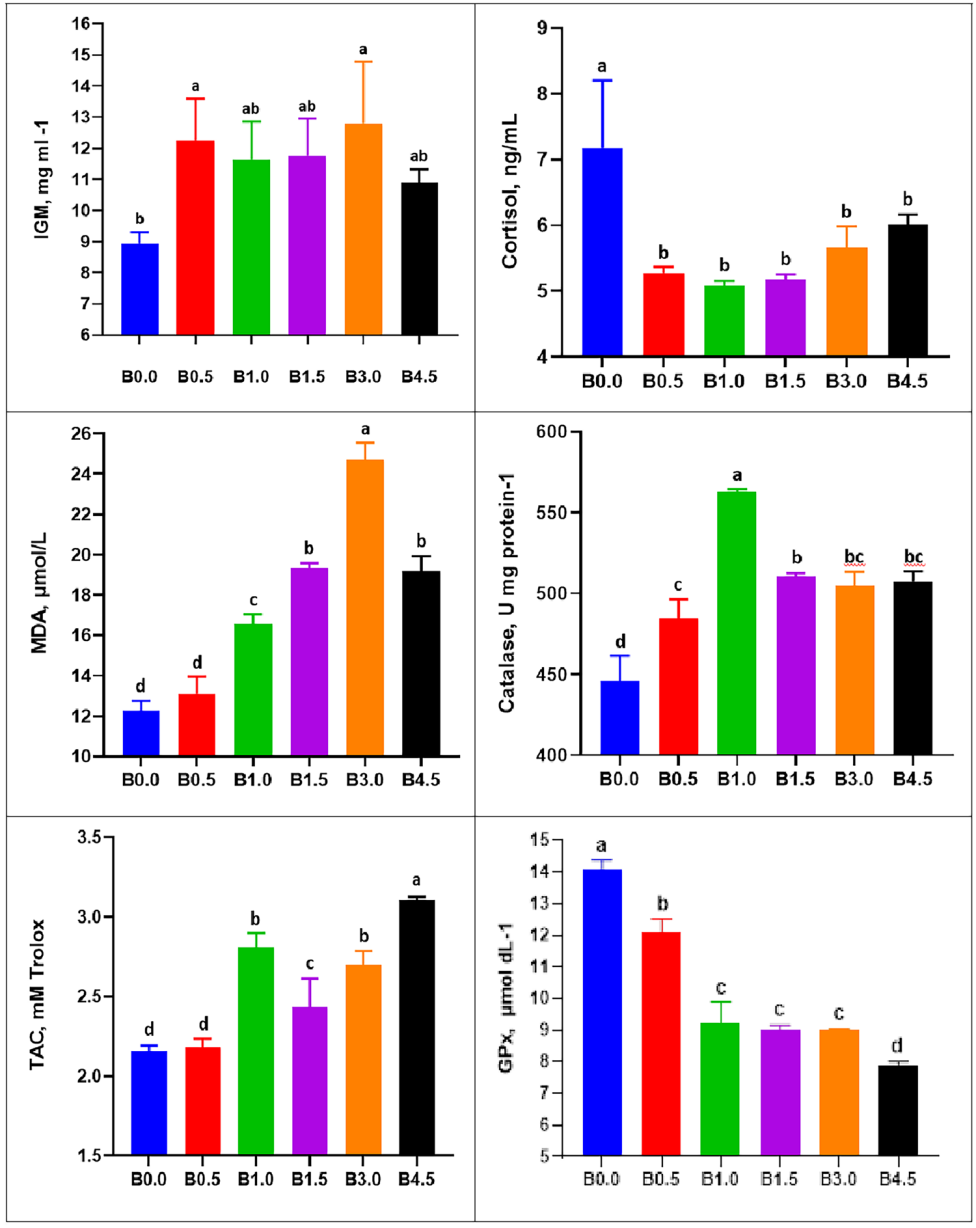
Immunity and antioxidant parameters of European seabass (*Dicentrarchus labrax* L.) fed with different levels of natural bentonite. Where B0 = 0% bentonite, B0.5 = 0.5% bentonite, B1 = 1% bentonite, B1.5 = 1.5% bentonite, B3.0 = 3.0% bentonite, and B4.5 = 4.5% bentonite.

Regarding the immune parameters of *D. labrax*, the BN-supplemented diets of fish had significantly (*P* < 0.05) higher levels (*P* < 0.05) of GLU (group B4.5), TP (groups B1, B1.5 and B3) and GLO (groups B0.5, B1, B1.5 and B3) (Table 7). Furthermore, an improvement in IgM level was observed in all groups supplemented with BN with a significant increase (*P* < 0.05) for the B.05 and B3 groups compared to the control (Fig. 2). Furthermore, an improvement in digestive enzymes (amylase and lipase) occurred in BN-fortified fish feed diets, as exhibited in Table 8. The highest amylase activity was recorded for fish in the B3 and B4.5 groups, while the highest lipase activity was reported for fish in group B1.5.

**Table 7:** Influences of dietary bentonite on serum immune parameters of European seabass (*Dicentrarchus labrax*).

Serum parameters	Treatments					
	B0	B0.5	B1.0	B1.5	B3.0	B4.5
Glucose	154.0 ± 11.0^bc^	129.0 ± 4.0^c^	173.0 ± 5.0^ab^	131.0 ± 3.0^c^	143.5 ± 9.5^c^	183.0 ± 6.0^a^
Total protein	3.82 ± 0.05^b^	3.99 ± 0.02^b^	5.05 ± 0.07^a^	5.02 ± 0.10^a^	4.88 ± 0.01^a^	4.35 ± 0.57^ab^
Globulin	1.65 ± 0.09^b^	2.74 ± 0.06^a^	2.90 ± 0.13^a^	3.19 ± 0.02^a^	3.08 ± 0.03^a^	2.56 ± 0.65^b^

The values with a different superscript in the same row are significantly different (*P* ≤ 0.05). Where B0 = 0% bentonite, B0.5 = 0.5% bentonite, B1 = 1% bentonite, B1.5 = 1.5% bentonite, B3.0 = 3.0% bentonite, and B4.5 = 4.5% bentonite.

**Table 8:** Influences of dietary bentonite on liver enzymes (AST, ALT, ALP) and digestive enzymes (lipase and amylase) of European seabass (*Dicentrarchus labrax*).

Treatments	Liver enzymes			Digestive enzymes	
	AST (U/l)	ALT (U/l)	ALP (U/l)	Lipase (U/l)	Amylase (U/l)
B0.0	240.5 ± 1.5^a^	38.0 ± 1.0^b^	2.17 ± 0.05^a^	35.5 ± 2.5^**b**^	29.5 ± 1.5^**c**^
B0.5	44.5 ± 2.5^e^	24.0 ± 1.0^c^	1.25 ± 0.08^c^	36.5 ± 2.5^**ab**^	32.5 ± 2.5^**bc**^
B1.0	82.0 ± 6.0^d^	25.0 ± 2.0^c^	1.79 ± 0.08^b^	42.0 ± 1.0^ab^	38.0 ± 3.0^b^
B1.5	80.5 ± 5.5^d^	23.5 ± 1.5^c^	1.83 ± 0.12^b^	43.0 ± 1.0^**a**^	33.5 ± 1.5^bc^
B3.0	174.5 ± 5.5^c^	39.0 ± 1.0^b^	1.81 ± 0.04^b^	42.5 ± 2.5a^b^	65.5 ± 0.5^a^
B4.5	202.5 ± 8.5^b^	56.5 ± 1.5^a^	2.15 ± 0.06^a^	40.5 ± 1.5^ab^	68.0 ± 3.0^a^

The values with a different superscript in the same column are significantly different (*P* ≤ 0.05). Where B0 = 0% bentonite, B0.5 = 0.5% bentonite, B1 = 1% bentonite, B1.5 = 1.5% bentonite, B3.0 = 3.0% bentonite, and B4.5 = 4.5% bentonite.

Regarding the activities of cortisol and antioxidants (CAT, TAC, MDA, and GPx), Fig. 2 shows that their concentrations were significantly affected by the addition of BN to the diet. Compared to the control group, fish fed BN-fortified diets at different concentrations showed a significant (*P* < 0.05) decrease in cortisol levels. Furthermore, the addition of BN to the diet made the CAT levels significantly (*P* < 0.05) higher in all treated groups compared to the control group. The addition of BN did not improve GPx levels, which decreased significantly (*P* < 0.05) compared to the B0 group. Supplementation of the *D. labrax* diet with BN improved TAC activity in all treated groups except for the B0.5 group which did not differ significantly from B0. However, with the exception of the B0.5 group, MDA levels increased significantly (*P* < 0.05) in the BN-treated groups compared to the control.

### Influences of dietary BN on the histology of the intestine and liver

**Figure 3:**
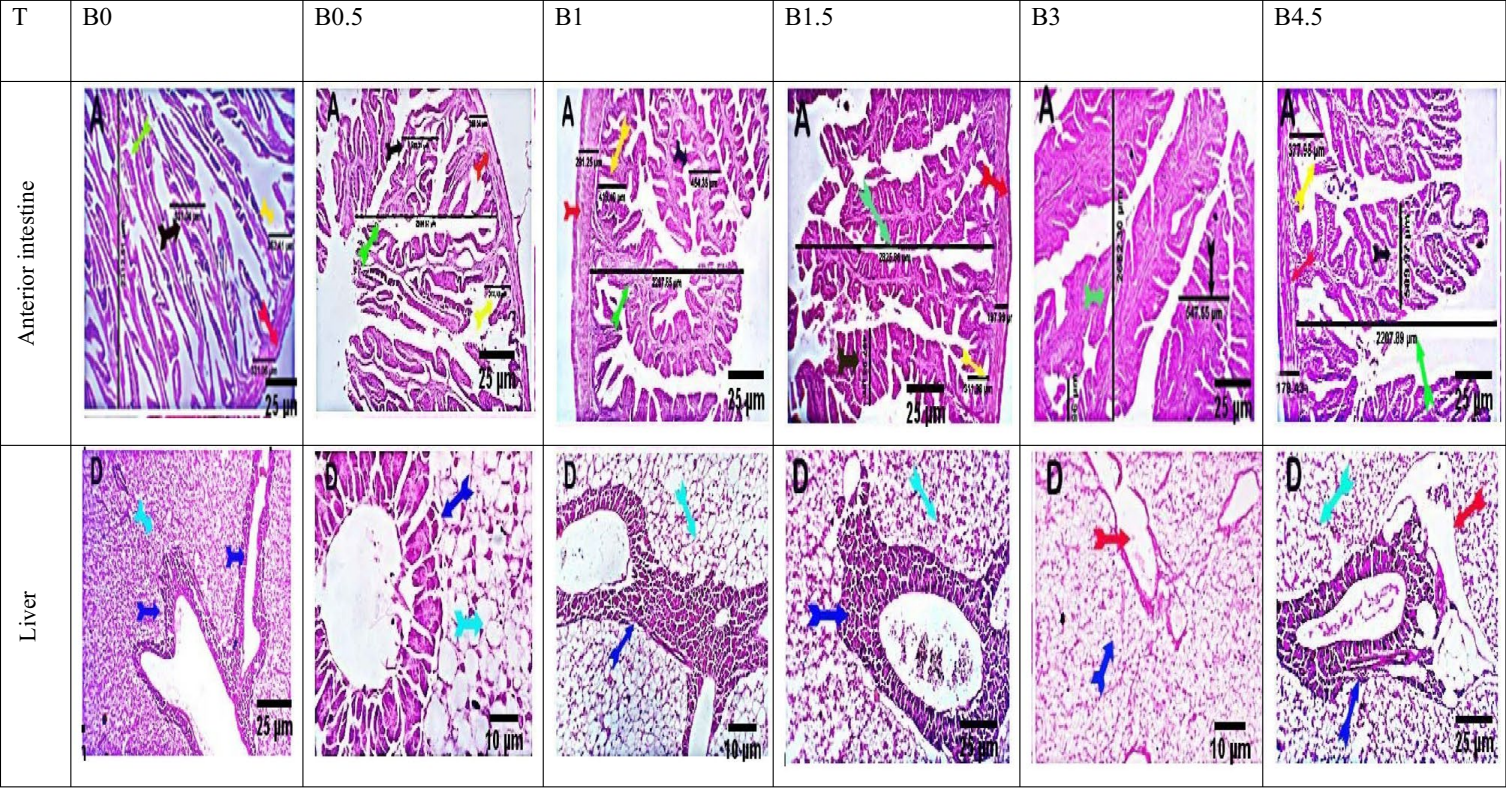
Photomicrograph of the anterior intestine and liver of European sea bass (*Dicentrarchus labrax* L.) fed raw bentonite at levels of 0, 0.5, 1, 1.5, 3, and 4.5% Per kg feed for 84 days. Where B0 = 0% bentonite, B0.5 = 0.5% bentonite, B1 = 1% bentonite, B1.5 = 1.5% bentonite, B3.0 = 3.0% bentonite, and B4.5 = 4.5% bentonite. H&E, Scale bars 25 um and 10 um. Group B0 demonstrates normal histological characteristics, including villi with branching (green arrow), normal villous length (green arrow), villous width (black arrow), crypt length (yellow arrow), and muscle coat thickness (red arrow) (red arrow). Group B0.5 demonstrates normal histological characteristics, including relatively short, branched intestinal villi (green arrow), normal villous breadth (black arrow), crypt length (yellow arrow), and muscle coat thickness (red arrow) (red arrow). There is an increase in the number of goblet cells (orange arrows), short, thick celli (brown arrows), and hepatocellular vacuolations (light blue arrows). Group B1.0 demonstrates normal histological characteristics, including villi with branching (green arrow), normal villous length (green arrow), villous width (black arrow), crypt length (yellow arrow), and muscle coat thickness (red arrow) (red arrow). There is a normal population of goblet cells (orange arrow), long, thick celli (brown arrow), and hepatocellular vacuolations (light blue arrow). Group B1.5 demonstrates normal histological characteristics, including relatively long, branched intestinal villi (green arrow), normal villous width (black arrow), crypt length (yellow arrow), and muscle coat thickness (red arrow) (red arrow). Increased goblet cells (orange arrow) and more active pancreatic acini are shown. Group B3 demonstrates normal histological features, including villi with branching (green arrow) and increased villous width (black arrow). The length of the crypt (yellow arrow) and thickness of the muscular coat (red arrow) appear normal. There is a modest amount of goblet cells (orange arrow), highly branching big intestine folds (orange arrows), and interlobular hepatic vascular dilatation (red arrow). Group B4.5 demonstrates normal histological characteristics, including relatively short branched intestinal villi (green arrow) and increased villous width (black arrow). The length of the crypt (yellow arrow) and thickness of the muscular coat (red arrow) appear normal. There are a moderate number of immune cells (yellow star) and periportal hepatic vascular dilatation (red arrow).

Different levels of BN supplementation in the diet of *D. labrax* had a positive effect and enhanced histological characteristics (Fig. 3). For all groups, microscopic examination of the intestine revealed normal histological characteristics with branched intestinal villi. Compared to the control group, the fish in group B1.5 exhibited more goblet cells, more active pancreatic acini, and relatively lengthy, branching intestinal villi. Furthermore, the fish-fed diet B1.5 had higher villi length values, while the fish-fed diets B3 and B0.5 had the highest villi width values, respectively, with no significant differences between them (Table 9). Furthermore, the B1 diet fed fish exhibited the longest crypts. Diets supplemented with BN at concentrations ranging from 0.5 to 3% improved villi length (B1.5 group), villi width (B3 group) and crypt length (B1 group) relative to the control diet (B0) (Table 9). Normal histological features were observed in the livers of the six experimental groups, with hepatocellular vacuolations in groups B0.5 and B1, interlobular hepatic vascular dilation in group B3, and periportal hepatic vascular dilation in group B4.5 (Fig. 3).

**Table 9:** Histology parameters of the anterior intestine of *Dicentrarchus labrax* fed diets supplemented with six levels of bentonite.

Histology parameters	Treatments					
	B0.0	B0.5	B1.0	B1.5	B3.0	B4.5
Villi length, µm	2310.3 ± 1.2^c^	2021.6 ± 2.9^e^	2250.6 ± 16.1^d^	2776.6 ± 32.2^a^	2586.3 ± 33.1^b^	2026.8 ± 97.3^e^
Villi width, µm	529.5 ± 1.1^a^	526.4 ± 3.6^a^	450.0 ± 3.1^c^	472.2 ± 18.3^d^	536.4 ± 7.2^a^	495.6 ± 9.2^b^
Crypt's length, µm	349.9 ± 1.6^c^	368.3 ± 5.4^b^	415.0 ± 2.3^a^	309.9 ± 4.1^e^	319.8 ± 11.3^d^	360.4 ± 9.2^b^
Muscular layer thickness, µm	324.8 ± 3.7^a^	253.3 ± 3.0^c^	284.0 ± 4.4^b^	191.9 ± 3.1^d^	164.9 ± 1.2^f^	170.4 ± 4.6^e^

Values are expressed as the mean ± SEM of three samples. Different letters in the same row indicate significant differences among the treatments (*P* ≤ 0.05). Where B0 = 0% bentonite, B0.5 = 0.5% bentonite, B1 = 1% bentonite, B1.5 = 1.5% bentonite, B3.0 = 3.0% bentonite, and B4.5 = 4.5% bentonite.

## Discussion

Due to the rapid global expansion of aquaculture to generate large amounts of animal protein and the scarcity of land and water resources, intensification of aquaculture operations is one of the viable options to meet the global demand for seafood (Kurian et al. 2020). The quality of the feed, the aquatic environment, and the culture system used directly affect the growth performance and health of cultured organisms^4,39^.

The present experiment demonstrates a clear improvement in water quality. The results for TAN and NH3 values in the six groups demonstrated that the addition of BN to the diets decreased TAN and NH3 excretion and that there was a direct correlation between an increase in BN of up to 1.5% in the diet and ammonia depletion. This finding is consistent with the findings of Ayoola^12^ who discovered that the addition of BN to the diets of African catfish (*Clarias gariepinus*) decreased the levels of ammonia in the rearing water. Unconsumed feeds contributed to an increase in bad water quality, but the high water stability of clay-supplemented diets maintained good water quality compared to the control diet. On the other hand, several previous studies^5–7,16,40,41^ reported that when BN was added to fish farming water, toxic compounds (TAN and NH3) were reduced and the rate of starch and protein degradation increased due to absorption and ion exchange. The adsorption process is the time during which solution molecules aggregate on the outer and/or interior surface of a porous substance^42,43^. Both absorption and ion exchange are diffusion processes, and combine for a uniform treatment known as the sorption process, which is the transfer of aqueous molecules into a solid mass^41,42^.

This decrease in NH_3_ could be due to the ion exchange process that takes place between BN molecules and organic compounds, during which ammonium is recovered and reused for other purposes, such as a N fertilizer^41^. Furthermore, BN, which is a silicate sheet clay with high cation exchange and ion adsorption capacity, has a higher selectivity for ammonium removal due to its large surface areas of a net negative charge; as a result, inorganic and organic cations (NH4^+^, Pb^+2^, Cu^+2^, K^+^, etc.) can be retained in the water^2,41,44^. The total cation exchange capacity of BN ranges from 40 to 130 meq/100 g^45^.

Preceding studies have shown that including clay minerals in the diet of fish improves growth, immunity, and disease resistance^11,15,46,47^. Compared to the control group in the present study, the inclusion of BN in the diet of *D. labrax* significantly increased growth indices and feed efficiency in the present experiment. The best indices favored the BN groups, as their results displayed a peak in group B 1.5, which had the highest FW, WG and SGR and the best FCR. The beneficial effects of dietary BN are consistent with previous studies on Nile tilapia *Oreochromis niloticus*^17^, African catfish, *Clarias gariepinus*^12^, rainbow trout, *Oncorhynchus mykiss*^11,15,46,47^, and hybrid grouper *Epinephelus fuscoguttatus* x *Epinephelus lanceolatus*^16^. In addition, Kurian et al.^48^ reported that the combination of Leucas Aspera, Oxy-cyclodextrin and sodium BN improved growth and digestive enzyme, preserved liver tissue, and stimulated the innate immunity of Nile tilapia. As a clay mineral, BN had a significant effect on the growth performance and FCR of *D. labrax*, indicating that the capacity of dietary BN to improve growth performance is dose dependent.

For marine species, the results herein are consistent with those of Arshad et al.^16^ who discovered that hybrid grouper fed a 1.5% sodium BN supplemented diet for 8 weeks exhibited significant growth and feeding utilization improvements, recording the highest weight gain and the best FCR (1.54). The scientists attributed these results to the fact that BN improved the usage of nutrients by slowing the transit of predigested feed through the intestines of fish. This resulted in a greater utilization of nutrients, especially protein, which led to greater growth^47^.

Current findings can be attributed to a variety of variables, (1) BN inclusion improved food intake by modifying conditions in the digestive tract, such as pH, buffer capacity, feed dilution, and osmotic pressure^21^; (2) BN consumption lowers or eliminates aflatoxin^49^, and its protective effects against toxicity are associated with toxin absorption^50^; (3) BN is an effective binding factor and plays a role in the absorption of heavy metal ions and mycotoxins from feeds^51^; and (4) as a clay mineral, BN stabilizes the intestinal barrier and is capable of absorbing pathogenic microbes, enzymes and toxins, making it a useful treatment for gastrointestinal diseases^52^. In the current experiment, compared to the B1.5 group, the B3 and B4.5 groups showed a significant decrease in SGR and FCR. High levels of BN inclusion resulted in a high viscosity of the food, decreased digestive enzyme mixing, increased endogenous nutritional losses, and increased the thickness of the whirlpool water stratum close to the mucus, which hindered the digestion process^53^. Furthermore, large quantities of BN may promote toxic fermentation in the fish intestines.

Blood quality measurements represent biochemical changes that occur in animals, revealing their metabolic and physiological status in general. Therefore, blood biochemistry measures are often used as diagnostic tools in biomonitoring, allowing the detection of pathophysiological changes attributed to nutrition^54^. The increase in the number of red blood cells (RBCs) in diets containing BN, particularly diet B1.5, is consistent with the findings of the researchers^16,55^. According to Radu et al.^55^ a high level of RBCs reflects a healthy physiological state, which is reflected in growth performance. However, in the present study, higher concentrations of BN (above 1.5%) resulted in a reduced content of RBC. This result is consistent with those of Arshad et al.^16^. According to Ivanc et al.^56^ a decrease in RBCs may indicate a disturbance in the consumption or quality of the diet in fish. The decrease in WBC of fish fed BN-enriched diets, particularly diet B1.5, is consistent with the findings of previous studies^16^. White blood cells (WBCs) are a heterogeneous class of nucleated cells that play a crucial role in phagocytosis and immunity, and therefore in defense against infection and foreign substances^57^. Increases in WBC counts may suggest a disturbance in the normally healthy immune system^58^. Similarly, Jawahar et al.^13^ observed that the increasing inclusion of zeolite as a feed additive led to an increase in white blood cell counts in several fish. Previous studies have proven the correlation between increased hemoglobin concentration and dietary BN addition, as this present study demonstrates^17^.

The decrease in serum CHO values in fish fed diets containing BN in the B.05 and B1.5 groups is a significant indicator of fish health. This is consistent with Karimi et al.^23^. The CHO and TG values obtained in the current study were remarkably similar to those seen in Montmorillonite-fed rainbow trout, the component that constitutes about 80% of the BN. Increased CHO (B3 and B4.5 groups) and TG (B1, B1.5, B3 and B4.5 groups) may be expressed as particularly elevated BN concentrations led to increased viscosity and thickness of the feed and thickness of the whipped layer close to the mucus, and could also cause baleful leavening in fish guts^53,59^. On the other hand, the addition of BN to the diet improved HDL levels (groups B3 and B4.5) and decreased LDL values (groups B.5 and B1.5) and their values obtained here were lower than those reported in the previous study on rainbow trout. As important diagnostic indicators, serum liver enzymes (AST, ALT, and ALP) and serum kidney indicators (CRE, UA, and urea) are frequently used to assess fish health and condition^60^. In the present study, fish fed the BN-supplemented diets had lower levels of urea (B1, B1.5, B3, and B4.5) and UA (B1 and B1.5) than fish fed the control and other treated diets. Furthermore, with the exception of the B4.5 group, an overall improvement in liver function occurred in fish of the BN-treated groups, particularly (B1.5), where lower enzyme levels were recorded. These enhancements can be expounded as BN has a detoxifying task that drives the absorption of venoms and harmful substances. Also, it has lubricity, moldability, low permeability, and high dry bond strength, making it an extraordinary substitute for adsorbents for dismissing toxins and contaminants^61^. This was consistent with the findings of previous studies that fish in good health condition had low levels of previous enzymes and vice versa^4,61^.

The importance of digestive enzymes in nutrient absorption and feed utilization is crucial. In line with the findings of Kurian et al.^48^, the current study showed a significant increase in the concentration of digestive enzymes (lipase and amylase). This improvement may be attributed to the ability of BN to remove harmful gases and toxins from the intestine^2,62^, which results in improved absorption through the intestinal tract^63–65^, leading to the increased metabolic activity of digestive enzymes.

Innate immunity is the major form of defense in fish. Lysozyme and immunoglobulins are two of the most commonly studied biomarkers of the innate immune response^66^. Existing research on the effect of BN on the immunological and antioxidant characteristics of *D. labrax* is extremely limited. Serum TP is a significant clinical indication of the nutritional state, stress level, health, and liver function of fish. Furthermore, serum TP contains nonspecific immunological factors, including immunoglobulins^64^. The TP values in the current study were almost identical to those obtained in the previous rainbow trout study^23^. This indicates superior immune system development in the BN-supplemented groups. Furthermore, similar to the present findings, serum TP levels of *Channa striatus* fed Zeolite-supplemented diets were markedly elevated^13^. Immunoglobulins or antibodies, which are important for adaptive immune responses, belong to the Ig superfamily^67^. IgM is the most dominant Ig in fish plasma and has been regarded as the oldest antibody class for a very long time. In this Ig-mediated humoral defense, the complement system is activated, viruses and poisons are neutralized, and pathogens are opsonized for phagocyte destruction^68^. In this study, diets supplemented with BN considerably increased seabass plasma IgM levels. The results of the previous parameters (TP and IgM) in the current study are very compatible with those of other published studies^4,69^. Cortisol has been proven to be a reliable stress biomarker and is considered the primary stress hormone^70^. In the current study, all diets supplemented with BN decreased cortisol levels compared to the control diet. Cortisol levels in fish are affected by feed type^71^, stocking density^72^, and water recirculation^73^. Significantly negative effects of the chronic increase in plasma cortisol on fish appetite, growth performance, condition factor, and feed conversion were reported^74^.

Fish have both enzymatic and nonenzymatic antioxidant defense systems that protect them from the effects of oxidative stress. Antioxidant defense systems contain antioxidant enzymes that defend against tissue oxidative damage^75^. In the current study, the activity of oxidative enzymes (TAC, CAT, and GPx) was assessed, with a substantial improvement in CAT and TAC. To assess the process of oxidative stress in an aquatic animal, CAT, TAC, and GPX are important relevant antioxidant factors that are palpably noted to provide defense against oxidative stress and distortions resulting from their interaction with fish exposed to various environmental contaminants, and their activity is evidence of a strong protection system in fish^61,75^. The increase in CAT is consistent with earlier studies on Nile tilapia^76^, and seabass larvae^75^, but the increase in TAC is consistent with other studies on Japanese seabass^69^. According to Gupta et al.^77^ fish treated with probiotics had higher levels of catalase and superoxide dismutase (SOD) activity. Higher CAT and TAC activities in BN-fed fish in the present study may indicate a healthy hepatopancreas with little damage. BN may also play an important role in the increased antioxidant capacity of seabass, as found by Abbas et al.^61^.

Growth, disease resistance, and food utilization are significantly influenced by the healthy development of fish internal organs, especially the gut and liver^4,65^. Light microscopy in this study showed that the intestine and liver of BN-treated *D. labrax* had normal and healthy morphology, and the B1.5 group showed the greatest improvement in both organs compared to the control group. The improved intestinal morphology, goblet cell abundance and microvilli length of seabass fed a diet supplemented with BN and normal liver tissue (B1.5) are in line with the outcomes of Abbas et al.^61^ who found that supplementing the Nile tilapia diet with BN significantly improved liver and kidney functions, as evidenced by a decrease in plasma values of liver enzymes and kidney damage indices. BN defends the liver and intestinal tract of fish by increasing the activity of the microintestinal flora, which aids in the absorption of nutrients, along with many inorganic and organic compounds in the digestive system^78^, boosting vitamin B and K synthesis and promoting bile acid and xenobiotic metabolism^79,80^. BN was shown to improve fish liver function^12,79^, and the hepatorenal function^61^.

## Conclusion

Many countries with lower standards for the quality of fish and/or aquafeed seek efficient and cost-effective feed additives to counteract the presence of various toxins and/or nutrient deficiencies in the feed ingredients they use. From this perspective, the search for natural, inexpensive, effective, and readily available feed additives is a challenge in these countries. In this study, six different levels of BN, which is abundant and inexpensive in Egypt, were evaluated as feed additives in seabass farming using underground saltwater. The results showed that the diet supplemented with 1.5% BN improved juvenile seabass growth performance, feed utilization, blood biochemical analyzes, enzyme activities, antioxidant activity, immune response, and liver and intestinal histology. Therefore, the recommended concentration of BN in the seabass diet formulation is 1.5%.

## Data Availability

All data generated or analyzed during this study are included in this published article.
